# A patient-centred and multi-stakeholder co-designed observational prospective study protocol: Example of the adolescent experience of treatment for X-linked hypophosphataemia (XLH)

**DOI:** 10.1371/journal.pone.0295080

**Published:** 2024-01-19

**Authors:** Vrinda Saraff, Annemieke M. Boot, Agnès Linglart, Oliver Semler, Pol Harvengt, Angela Williams, Karen M. A. Bailey, Fiona Glen, Elin Haf Davies, Sue Wood, Stephen Greentree, Angela J. Rylands

**Affiliations:** 1 Department of Paediatric Endocrinology and Diabetes, Birmingham Women’s and Children’s Hospital NHS Trust, Birmingham, United Kingdom; 2 Institute of Applied Health Research, University of Birmingham, Birmingham, United Kingdom; 3 University Medical Center Groningen, University of Groningen, Groningen, The Netherlands; 4 Assistance Publique Hôpitaux de Paris, Université Paris Saclay, Bicêtre Paris-Saclay Hospital, Le Kremlin Bicêtre, France; 5 Department of Pediatrics, Faculty of Medicine and University Hospital Cologne, University of Cologne, Cologne, Germany; 6 XLH Belgium (Belgium XLH Patient Association), Waterloo, Belgium; 7 Kyowa Kirin International, Marlow, United Kingdom; 8 OPEN Health, Marlow, United Kingdom; 9 Aparito, Wrexham, United Kingdom; Federico II University, ITALY

## Abstract

The importance of patient centricity and keeping the patient at the heart of research design is now well recognised within the healthcare community. The involvement of patient, caregiver and clinician representatives in the study design process may help researchers to achieve this goal and to ensure robust and meaningful data generation. Real-world data collection allows for a more flexible and patient-centred research approach for gaining important insights into the experience of disease and treatments, which is acutely relevant for rare diseases where knowledge about the disease is more likely to be limited. Here, we describe a practical example of a patient-centric, multi-stakeholder approach that led to the co-design of a prospective observational study investigating the lived experience of adolescents with the rare disease, X-linked hypophosphataemia. Specifically, we describe how the knowledge and expertise of a diverse research team, which included expert physicians, research and technology specialists, patients and caregivers, were applied in order to identify the relevant research questions and to ensure the robustness of the study design and its appropriateness to the population of interest within the context of the current clinical landscape. We also demonstrate how a structured patient engagement exercise was key to informing the selection of appropriate outcome measures, data sources, timing of data collection, and to assessing the feasibility and acceptability of the proposed data collection approach.

## Introduction

Patient-centred medicine development, involving patients and health service end-users, is now considered to be integral to the development of new treatments [1]. With the existing framework of medicine development being criticised for failing to sufficiently focus on patients, the eventual recipients of medicines [2–5], it is widely acknowledged that the patient should be placed at the centre of research design [6]. Multistakeholder co-design of research that includes patients and/or their caregivers, treating physicians and methodology specialists may be more resource intensive, but it not only offers an important opportunity to amplify the patient voice in a robust and structured manner but also ensures that real-world observational studies address questions of significance to those who will be impacted by or need to make decisions based on the study’s findings [7, 8].

International initiatives and consortia [9] have produced best practice guidance to guide patient centricity in research and to facilitate the implementation of patient-relevant outcomes, according to recommended and robust methodology [10–13]. It is also common for scientific journals and ethics committees to require evidence of patient involvement in research approaches prior to approving content. Furthermore, it has been suggested that engaging patients in the study design process may lead to later efficiencies; for example, by reducing the need for protocol amendments and/or improving rates of enrolment, adherence or retention [14].

Not only are patient-centred and co-design approaches growing in importance, real-world evidence continues to attract interest for the evaluation of new medicines [15, 16]. Alternative methods of data collection that extend beyond the randomised controlled trial (RCT) are being adopted more readily, such as registries, prospective observational studies, mixed-methods research and qualitative interviews [8]. Although the RCT remains the gold standard for establishing treatment efficacy and safety for regulatory purposes, an understanding of how patients experience their disease and treatments in real life is critical to continue to gather evidence of the value of medicines. Real-world data capture is particularly important in rare diseases, where there may be little or no published information on the natural history or course of the disease. The capture of patient experience data with sufficient rigour therefore allows for a better understanding of the real world and the patient-specific impact of new therapies [17].

**Fig 1:**
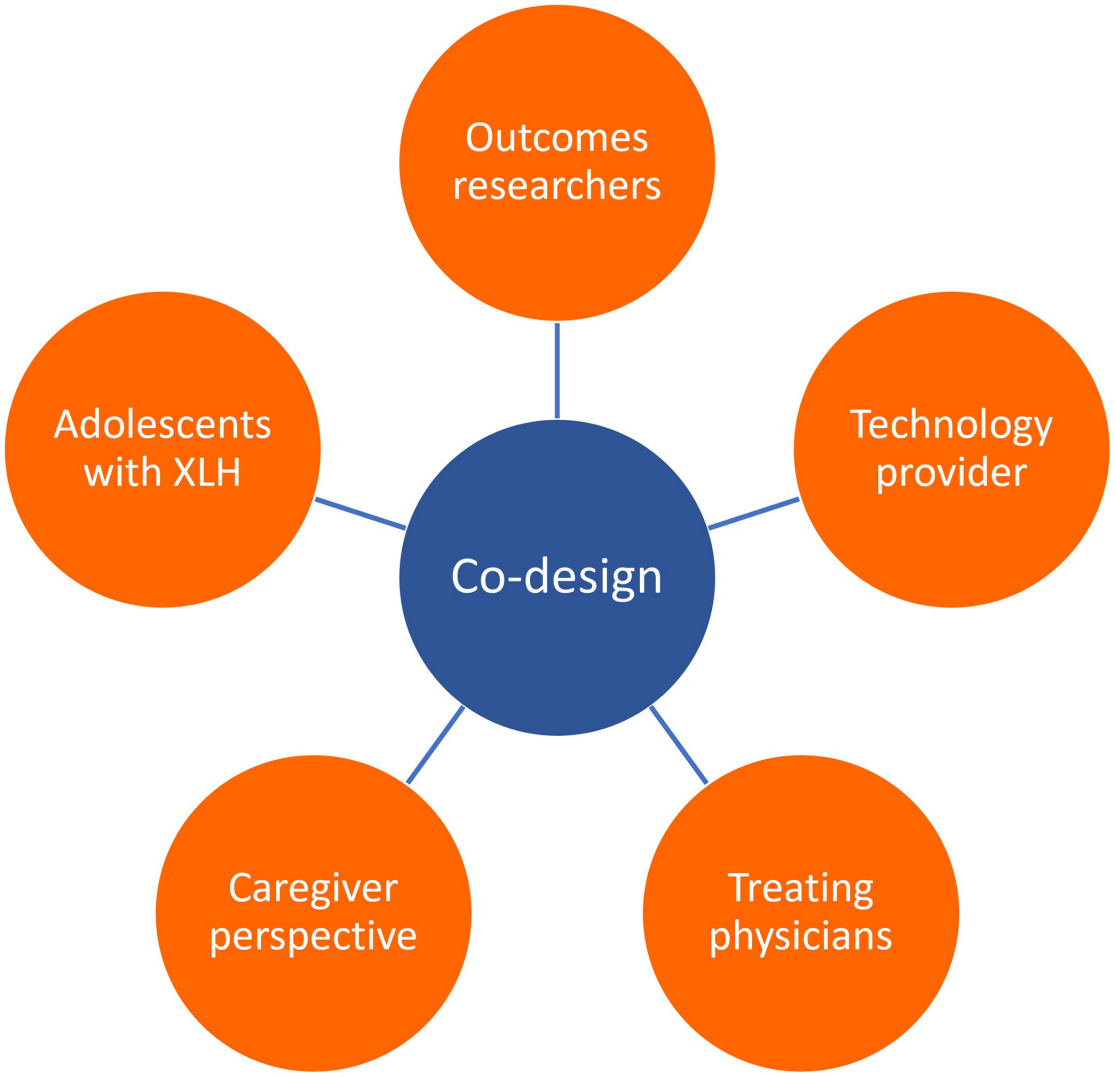
Overview of stakeholders involved in co-design of mixed-methods real-world study about the lived experience of adolescents with XLH at the end of skeletal growth.

Given this evolving research landscape, case examples of patient-centric, co-design approaches become increasingly noteworthy, particularly in the rare diseases. To that end, this article describes an example of a patient-centric, multistakeholder co-design approach for a prospective observational study investigating the lived experience of adolescents with a rare disease, X-linked hypophosphataemia (XLH) (NCT05181839). Our approach involved the early partnership of a diverse research team to inform the research questions and methodology. The variety of perspectives were gathered from relevant stakeholders, including patient-centred outcome research specialists, adolescents with XLH, a caregiver of an adolescent with XLH, expert physicians from across Europe and specialist technology providers (Fig 1). The multidisciplinary input to the research design was intended to enable the generation of robust, meaningful and patient-centred outcomes for this rare disease population.

We took a stepwise approach to developing and finalising the research study in adolescents with XLH, which started with defining the research questions and methodology (*Section 1*). Our description of the final study design and the multi-stakeholder involvement in each subsequent component is presented in the following sections, structured according to the PICOT framework: 2.) *Population (P)*, 3.) *Intervention (I)*, 4.) *Comparison (C)*, 5.) *Outcomes (O)* 6.) *Time / duration (T)* [18, 19].

## Materials and methods

### Defining the research questions and methodology

Our overall research aim was to understand the lived experience of adolescents with XLH at the end of skeletal growth, a crucial phase during the adolescent’s transition to adulthood. XLH is a rare, genetic, life-long disease that is associated with progressive accumulation of multiple musculoskeletal features that evolve across the patient’s lifetime [20–23]. Some knowledge of the disease burden for children and adults exists [24–27]. However, data are limited for adolescents with XLH. Furthermore, medical management of adolescents varies between countries [28]. For these reasons, adolescents’ experiences of XLH and the impact of treatment at the end of skeletal growth were considered to be an evidence gap worth investigating. Traditionally, treatment for children and adolescents with XLH has been oral phosphate and active vitamin D analogues; however, in February 2018, burosumab, a recombinant human monoclonal immunoglobulin G1 antibody that inhibits fibroblast growth factor 23 (FGF23), received European Medicines Agency approval for individuals aged 1 year or older with radiographic evidence of bone disease and adolescents with growing skeletons [29]. This approval was subsequently expanded in September 2020 to include older adolescents who had stopped growing, as well as adults, offering a new treatment option targeting the underlying pathophysiology of XLH [30]. Given the changing treatment landscape and known variation in treatment pathways for adolescents with XLH across different geographical locations, the research team acknowledged the importance of studying the experiences of adolescents in relation to burosumab treatment as part of the study.

As well as a lack of data on adolescents’ experiences, very little is known about the caregivers of adolescents with XLH during the period of ending skeletal growth. Although there is some literature on the experience of caregivers of people with other rare bone disorders [31–34], there is a dearth of literature about the experience of caregivers of adolescents with XLH, particularly relating to during the period of ending skeletal growth. An understanding of the caregiver experience may allow for increased knowledge of the impacts of caring for an adolescent with XLH, which in turn may identify the wider support requirements of caregivers. Hence, the main research questions to investigate were defined as:

**What is the experience of adolescents with XLH as they approach the end of skeletal growth while receiving treatment with burosumab**?

**How do the experiences of adolescents with XLH vary in the 6 months following end of skeletal growth according to treatment received**?

**What is the experience of caregivers at the time that the adolescent with XLH reaches the end of skeletal growth**?

Next, the most appropriate research methodology to address the research questions was identified as a mixed-methods approach, which is the combination of quantitative and qualitative data for the purpose of increasing the breadth and depth of understanding [35]. It is most suitable when a quantitative or qualitative methodology is inadequate by itself to address the study purpose [36]. When considering the lived experience of XLH, it was apparent that quantitative data on symptoms, activity, duration and intensity, alongside pharmacodynamics data, would illuminate disease impact over the study period, whereas qualitative data via interviews would complement and expand understanding of the quantitative findings. A more complete understanding of the lived experience of adolescents during the study period would therefore rely on the totality of evidence derived from both quantitative and qualitative data.

Furthermore, the mixed-methods approach is underpinned by a pragmatic research paradigm, which proposes that researchers adapt their methodological approach for the particular problem being investigated [35]. Importantly, assessment of individual experience is vital, whilst maintaining flexibility to embrace both objective and subjective knowledge [37, 38]. Given the rare nature of XLH and limited available knowledge about the treatment-related experiences of adolescents in particular, a degree of flexibility was needed and therefore a mixed-methods design was considered to be the most appropriate choice.

An observational, prospective design was selected to examine the lived experiences of adolescents during the period occurring immediately before and after the end of skeletal growth and to describe any treatment-related variation in a real-world clinical setting. A fully integrated, convergent mixed-methods design was thus deemed to be optimal for addressing the research questions, with both qualitative and quantitative data collected separately throughout the study and later merged for an overall interpretation [39].

### Study population (P) & study setting

#### Study population

The study population is that which best informs the research question, ensuring rich and relevant data, as well as enabling transference of the study findings to similar contexts [40]. Following discussion with treating physicians, the target population was defined as adolescents aged 12–⁠⁠17 years with a confirmed diagnosis of XLH (documented diagnosis of XLH in medical records, and evidence of at least one of the following: hypophosphataemia and/or impaired phosphate reabsorption due to elevated FGF23; PHEX mutation) who had been treated with burosumab for at least 12 months and were, in the opinion of their treating physician, approaching the end of skeletal growth (based on the treating physician’s routine growth tracking methods). Adolescents who were non-adherent to treatment, defined as missing ≥2 injections of burosumab in the previous 12 months, and those scheduled for orthopaedic surgery during the study, were considered to be important to exclude as these criteria could bias study outcomes.

A sample size of up to 30 adolescents was considered to be sufficient for this mixed-methods study, which is not hypothesis testing [41, 42]. Although some heterogeneity is expected, recruiting up to 30 subjects should represent the population sufficiently to allow for analyses according to treatment after the end of skeletal growth. This sample size was also considered to be feasible from a practical perspective, following consultation with potential participating specialist paediatric centres and expert treating physicians, in terms of the likely numbers of eligible adolescents at those centres. Purposive sampling was deemed to be appropriate, whereby physicians would use their knowledge to identify adolescents that met inclusion criteria [41]. This approach was considered to be feasible, as well as appropriate, to the research question and was expected to generate data that was generalisable to similar contexts.

Caregivers will be included in the study if they are a parent or guardian who provides day-to-day support or care for the adolescent with XLH who is taking part in this study. The caregivers in the sample will be interviewed about their experiences and support needs as a principal caregiver of an adolescent with XLH. It was determined that a sample of 15 caregivers would likely be more than sufficient to reach theme saturation, considering the homogeneity of the data to be collected [43]. To ensure sufficient geographical diversity, at least three representatives from each of the participating countries will be targeted for participation.

In this real-world study, a specific definition for the “end of skeletal growth” was not applied. Instead, the methods used to determine this stage aligned with local practices, which may vary across countries and among clinicians. However, clinicians were expected to document their approach to assessing the end of growth at the time of enrolment. This assessment included factors such as projected index dates, confirmed index dates, specific growth velocity thresholds, final height measurements or imaging results. Additionally, it is important to note that the prediction of “4 weeks before the end of skeletal growth” was determined by clinicians.

#### Study setting

The research literature indicates that the study setting should be accessible, appropriate for recruitment of the study population, and have staff at the study centre who are interested in and able to support the conduct of the research project [41]. Well-established specialist centres in the UK and across Europe that treat adolescents with XLH with burosumab were deemed to be suitable for inclusion and were subsequently approached by the study sponsor. An important consideration for centre inclusion was their potential to recruit enough eligible participants. The lead physicians at these specialist centres were informed of the purpose of the study, its methodology and the mutual role that centres would play in study design, conduct, analysis and reporting.

### Intervention

At the time of designing the study, burosumab had been studied in pivotal RCTs for ages 1–12 years, as well as in adults (aged 18–⁠65 years) [44–48]. The initial indication for burosumab in Europe (February 2018) was for the treatment of XLH in children aged 1 year and older with growing skeletons and radiographic evidence of bone disease. The indication was extended in September 2020 for the treatment of XLH in children and adolescents aged 1–17 years with radiographic evidence of bone disease, and in adults, thus enabling adolescents to be treated after the end of skeletal growth [30]. As this is a real-world observational study, the decision to continue burosumab will depend on reimbursement approvals in the different countries, with some patients required to discontinue treatment with burosumab at the end of skeletal growth and instead be offered alternative treatment, such as oral phosphate and active vitamin D.

### Outcomes

Appropriate outcomes relevant to the population need to be identified to sufficiently answer the research questions. In other words, ***what*** data should be collected (i.e. the **data variables**), ***how*** should the data be collected (i.e. the **data sources**), and ***when*** should data be collected to be most informative but also not overburden the study participants (i.e. the **timing**).

Data collection from medical records would be required to understand the characteristics of this population and key pharmacodynamics and clinical information relating to the XLH disease course. However, for meaningful insights into the experiences of adolescents with XLH, the majority of the data should be derived directly from the patient. To assist with this aspect of the study design, adolescents with XLH were invited to participate in a structured patient engagement exercise to inform the selection of appropriate outcome measures, data sources and timing, and to assess the feasibility and acceptability of the proposed method of data collection. Principal investigators would not have access to data to identify individual patients during or after data collection. The patient advocacy group (PAG) XLH UK was approached to help with recruiting adolescents with XLH to participate in a 60-minute, one-to-one telephone interview with experienced interviewers from OPEN Health. A structured interview guide was developed with input from expert physicians to gather insights into adolescents’ experiences of living with XLH, their treatment and their opinions of the proposed study. Four adolescents consented to participate in the interviews and provided insights into the selection of outcomes and data sources in the study [49].

The interviews verified insights from clinical trials that symptoms of pain, stiffness and fatigue were important to measure and likely to be impacted by treatment. The physician experts reported that it would be relevant to capture the location of pain and use of analgesia, so these outcomes were also incorporated into the study design. The collection of data before and after the end of skeletal growth would then enable an evaluation of any changes in symptoms depending on the treatment regimen received after the end of skeletal growth.

In the interviews, adolescents described how their ability to participate in social and leisure activities was affected by their symptoms (S1 Fig). Participants reported being slower at keeping up with their friends; for example, when climbing stairs, catching the bus and during physical education at school. They also stated that, although they felt more tired/fatigued than their friends and found activities more difficult, they continued nonetheless to take part in them. Overall, the discussion suggested that there was a significant interplay between physical activity levels and symptoms of XLH (such as pain, stiffness and fatigue), to warrant monitoring activity closely in order to interpret changes.

**Fig 2:**
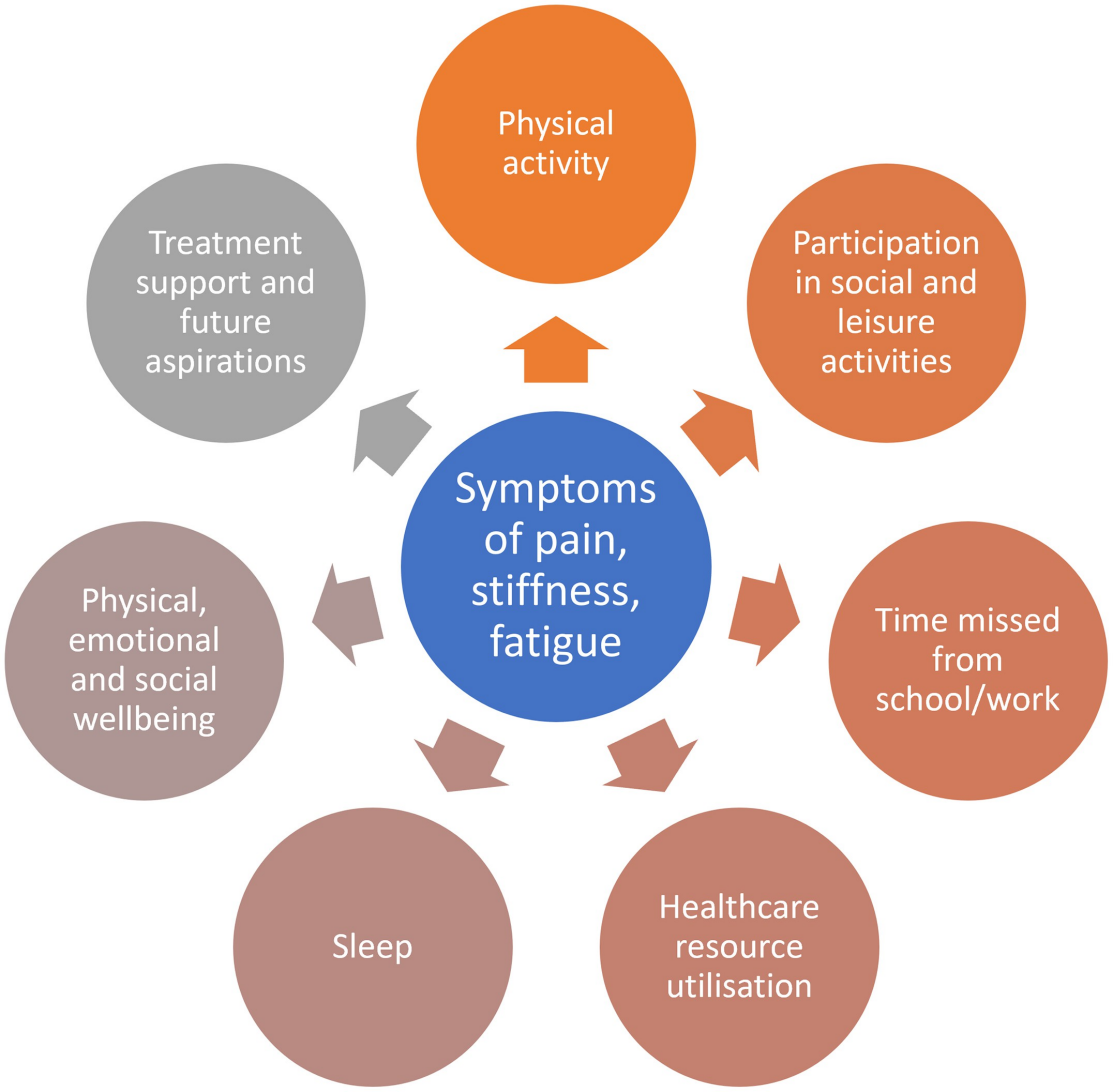
Concepts derived from patient interviews and following discussions with the research team.

To capture data on the duration and level of physical activity, as well as their participation in social and leisure activities, a wearable device was used, as well as collecting information on their participation in social and leisure activities. To further understand any wider impact of symptoms, the research team considered it appropriate to collect information on any time missed from school/work, as well as utilisation of healthcare resource (Fig 2).

The adolescents did not consider that there was a notable impact on sleep quantity or quality. However, the view of the expert physicians consulted on the study design was that sleep may be an interesting variable to measure, particularly given that this is something anecdotally reported as being affected due to levels of pain experienced (Fig 2). Given that the subjects would be wearing a device to capture activity levels in the daytime, and such devices have been used to collect data in the night, the research team proposed that participants should be given the option to wear the device at night to gain a proxy measurement of sleep quantity and quality activity data. Qualitative interviews would also provide another opportunity for adolescents to express their own views on sleep quality.

In addition, the team considered measuring the overall physical, emotional and social well-being of the subjects, with a general health-related quality of life outcome (HRQoL) measure (Table 1 and Fig 2). The EuroQol-5 dimensions-Youth (EQ-5D-Y) was chosen by the study team, as a short, widely used and well-validated HRQoL tool for the youth age range [50, 51].

**Table 1:** Overview of data collection schedule for quantitative and qualitative data relating to XLH symptoms and activity levels.

Topics	Endpoints	Source	Before end of skeletal growth^a^ (4 weeks)			After end of skeletal growth^b^ (26 weeks)		
			Daily	End of each week	At start of 4-week period	Daily for 1 week every 4 weeks	End of week for other study weeks	End of post-index period (Weeks 21–25)
**Pain**	• Worst pain	App						
	• Location							
	• Use of analgesia		X			X	X	
**Stiffness**	• Worst stiffness	App	X			X	X	
**Fatigue**	• Worst fatigue	App	X			X	X	
**Physical activity**	• Daytime duration	Wearable	X			X		
	• Daytime intensity							
**Social and leisure activities**	• Participation in social & leisure activities	App		X			X	
**School/work**	• Missed time	App		X			X	
**HCRU**	• Scheduled and emergency appointments	App		X			X	
**HRQoL**	• EQ-5D-Y	App			X			X
**Sleep**	• Duration	Wearable						
	• Quality		X			X		
**Adolescent experience**	• Lived experience	Telephone interview			X			X
**Caregiver experience**	• Identifying support needs	Telephone interview						X

EQ-5D-Y: EuroQol-5 dimensions-Youth; HCRU: Healthcare resource use; HRQoL: Healthcare-related quality of life; XLH: X-linked hypophosphataemia.

^a^Data will be collected prospectively for a 4-week period (as agreed in advance with the adolescent) to occur 4–12 weeks prior to the predicted date of end of skeletal growth.

^b^Data will be collected prospectively for 26 weeks from the confirmed date of end of skeletal growth (index date).

Given the nature of the selected endpoints and the age of the study participants, the research team considered that the outcomes could be measured using a bespoke designed smartphone app (Atom5^™^; Aparito, Wrexham, Wales, UK), as well as a wearable device (Garmin Vivosmart4^®^; Garmin, Ltd., Olathe, KS, USA). During the patient engagement exercise, adolescents were asked for their opinions about the use of smartphone apps and wearable devices (e.g. commercially available fitness watches) for data collection. All of the adolescents verified that they would be happy to input data via a smartphone app and they also expressed openness to wearing a wrist-based fitness device to track activity during the day, with the majority being happy to use this at night. This feedback endorsed the wider research team’s view around the wearable being an optimal and non-invasive tool for capturing data on physical activity levels and for sleep monitoring.

Finally, to collect qualitative data on the experiences of adolescents that would sufficiently complement and expand understanding of the quantitative findings, the research team included two one-to-one interviews with the adolescents before end of skeletal growth and at 6 months following end of skeletal growth as part of the overall study design. Interviews, based on a semi-structured discussion guide, would allow for an in-depth understanding of symptoms and an exploration of the social and emotional impacts of experiences, coping strategies and future aspirations, and provide further context to questions that are being asked during the app-administered surveys. This particular point was highlighted as being important by our caregiver member of the research team who was able to verify each of the outcomes identified by the adolescents from a patient and caregiver perspective.

By scheduling an interview at the end of the study, it would be possible to explore changes in symptoms and experiences over time and to examine the relationship between these changes on social and emotional experiences. In addition to interviewing adolescents with XLH, a separate interview with the adolescent’s caregiver during this same period following end of skeletal growth will provide an opportunity to gain insight into caregiver experiences.

### Time

Our patient engagement exercise also allowed for probing about the feasibility and acceptability of the overall timing of the study. The adolescents stated that a 12-month study was acceptable. All the adolescents stated that they would not expect any compensation for taking part in the study, but monetary vouchers would be welcomed, if offered.

Physicians with experience of treating adolescents with XLH advised that, due to the standard frequency of healthcare follow-up appointments, a period of up to 6 months was needed to identify, recruit and train participants on the study requirements, and to enable the collection of at least 4 weeks of prospective data before the end of skeletal growth experience. Furthermore, adolescents would need to be followed for at least 6 months after the confirmed date of the end of skeletal growth to ensure that any treatment-related effects had time to appear.

Another important consideration was the data collection schedule. Due to a lack of any previous studies, it is unclear how the presentation of symptoms of XLH around the time of end of skeletal growth may vary both within and between adolescents. As such, the research team considered it valuable to collect data for 4 weeks before the end of skeletal growth. In the interviews, the adolescents indicated that daily data collection using the app and wearable device for a defined period was considered to be acceptable (Table 1). Similarly, the pattern of change in symptoms and impact after end of skeletal growth is currently unknown. To provide a detailed picture of change without over-burdening the subjects, the data collection was scheduled to be periodic over 6 months (Table 1). During the interviews with the adolescents, they indicated that they would be happy to report on symptoms every day, but most preferred more detailed questions to be reported monthly, hence not overburdening them with too many questions on a daily basis, so these insights were implemented into the overall methodology.

### Patient engagement

As this study would require long-term engagement from the young participants, it was important to ensure that participants would be sufficiently supported throughout the study. In order to facilitate engagement and to minimise burden, a number of additional measures were put in place; for example, reminder notifications and features within the smartphone app to enable participants to request study-related support. Regular quarterly updates and newsletters will be provided and copy editors will be consulted to ensure that the language used in all study materials is accessible and appropriate for the age group.

### Data analyses

This is a mixed-methods research study, whereby descriptive analyses of quantitative data (medical records, app, wearable device and EQ-5D questionnaire) will be synthesised with qualitative data (interviews) to describe the lived experience of XLH for adolescents at the end of skeletal growth in the pre-index and post-index periods [39]. To address missing data, there will be no imputation for symptom diary and wearable parameters, with a threshold of 3 days’ data in a week being required to calculate a weekly average. A sensitivity analysis will be performed using a minimum of 4 days required to calculate a weekly average score for symptoms and wearable parameters.

Distributions and descriptive statistics of central tendency (medians and arithmetic or geometric means) and dispersion (standard deviation, interquartile range [IQR], range) will be presented for quantitative variables. Any categorical variables will be described with frequencies and percentages; where appropriate, distributions, modes, medians, IQR and range will be reported. Daily measurements (e.g. pain, stiffness and fatigue measurements collected using the app) will be summarised per adolescent for key time-points pre- and post-index, to aid interpretation of change post-index and individual data trends visualised over time. EQ-5D-Y scores will be converted to a single utility value during data analysis. Visual Analogue Scale and scores for each of the five domains assessed in the EQ-5D-Y (mobility, self-care, usual activities, pain/discomfort and anxiety/depression) will also be summarised separately. Statistical analyses will be carried out using Stata version 14.2 (StataCorp LLC).

Qualitative/thematic data analyses will be conducted using the Framework approach, engaging the use of matrices to manage data, allowing for easy movement across the whole dataset in order to explore patterns in the themes across different groups of participants, as well as trajectories within individual participants [52]. Analyses on patient data will be conducted cross-sectionally at baseline and for the post-index interview to allow for analysis between individuals, in addition to being analysed longitudinally for each individual, in order to capture a narrative for each adolescent and the trajectory of change. Carer data will be analysed separately.

Recruitment began on 24 November 2021 and is ongoing. Data collection is expected to end in 2023. The first data entry from medical records was on 24 November 2021. Data analysis is planned to commence in 2024.

## Discussion

Here, we have outlined an example showing how multistakeholder engagement, including (outcome) research specialists, and partnering with other relevant stakeholders, including adolescents with XLH, a caregiver and expert physicians from specialist centres across Europe, and technology providers, can facilitate the design of a patient-centric study in rare disease. The approach is aligned with a number of international initiatives aiming to engage patients in study design and to keep the patient voice at the centre of research and throughout the medicine lifecycle [10–13, 53]. The pragmatic, mixed-methods design has also been endorsed by the International Rare Disease Research Consortium as the “best fit for rare diseases” [54] and has been used successfully within clinical trials and wider studies investigating experiences of therapy [55, 56].

The study’s inherent flexibility may help to overcome some of the challenges associated with conducting studies in patients with rare disease and offers a more holistic insight into the experiences of patients that would not be as possible using the more traditional RCT design. Furthermore, our chosen approach of merging qualitative and quantitative data [39] after initial analysis will allow for further exploration of individual case studies and will help to capture any wider reasons (beyond treatment) that might have influenced any changes in symptoms or experiences observed over the study period.

It is also apparent that early engagement with patients who are representative of the target population to input on study design is possible and will improve feasibility of the study. We have demonstrated how a patient engagement approach can be successfully implemented in studies involving adolescents as well as adults, and that doing so can help the research team to select the most appropriate methodological approaches and tools for the intended study population to maximise data quality and meaning. This exercise verified that a mixed-methods design could be used to evaluate symptoms in this specific age group in a small group of patients with a rare disease.

Our study focused on the care of adolescents with XLH receiving burosumab treatment in specialist centres. XLH is usually diagnosed by a paediatrician or paediatric endocrinologist [57]. Knowledge and treatment of XLH is generally limited to specialist centres, with the condition demanding care from multidisciplinary teams [29], including endocrinologists, nephrologists and geneticists [58]. Furthermore, burosumab is increasingly becoming the standard of care, evidenced by its reimbursement in many European countries and its approval for use in children prior to reaching the end of skeletal growth [28, 29].

The multistakeholder approach described here will not be restricted to the study design phase of this project; indeed, consultations with a wider study steering committee, including a patient, caregiver and physicians, are planned throughout the study duration to review progress and ultimately ensure that outputs are robust, meaningful and accessible to those living with XLH, as well as the wider clinical community. Lay summaries of the study report and any resulting publications will also be shared with the study participants.

There are some limitations associated with our approach. Although critical to informing our design, the participants in the patient engagement exercise were recruited via a UK-based PAG and so it is unclear whether these views are representative of adolescents with XLH treated in other countries. However, our multistakeholder approach, involving patients, caregivers and healthcare professionals, does require more resources, but it significantly enriches our understanding of XLH. This collaborative approach ensures a comprehensive perspective, facilitates data-sharing, and promotes interdisciplinary insights, thus advancing knowledge in underexplored areas. Additionally, we must acknowledge our reliance on clinicians’ judgement, and practices for assessing "end of skeletal growth" introduces inherent variability across assessments. The complexity of skeletal maturation, influenced by genetics, environment and clinical factors, makes standardisation challenging [59]. Given these complexities, further research is needed to establish more precise and standardised methods for assessing skeletal growth endpoints.

With advancement in technology and integrated data systems, we anticipate that wearable technology linked with patient self-reports, interview and medical record data will enable better characterisation of adolescents with XLH and aid physicians in clinical decision-making.

Our multistakeholder engagement approach to designing this research study has facilitated the robust design of this patient-centric study in a rare disease and offers an inclusive approach, which could potentially be used as a possible template for future patient-centric study designs in other rare disease areas.

## Supporting information

S1 Fig Example quotes from patient engagement exercise. (TIF)

S1 File Plain language summary. (PDF)

S1 Checklist Inclusivity in global research. (DOCX)
