# Supplementary figures and images for: A patient-centred and multi-stakeholder co-designed observational prospective study protocol: Example of the adolescent experience of treatment for X-linked hypophosphataemia (XLH)
Source: PLoS One. 2024 Jan 19;19(1):e0295080. doi: 10.1371/journal.pone.0295080 (PMC10798437; doi:10.1371/journal.pone.0295080)

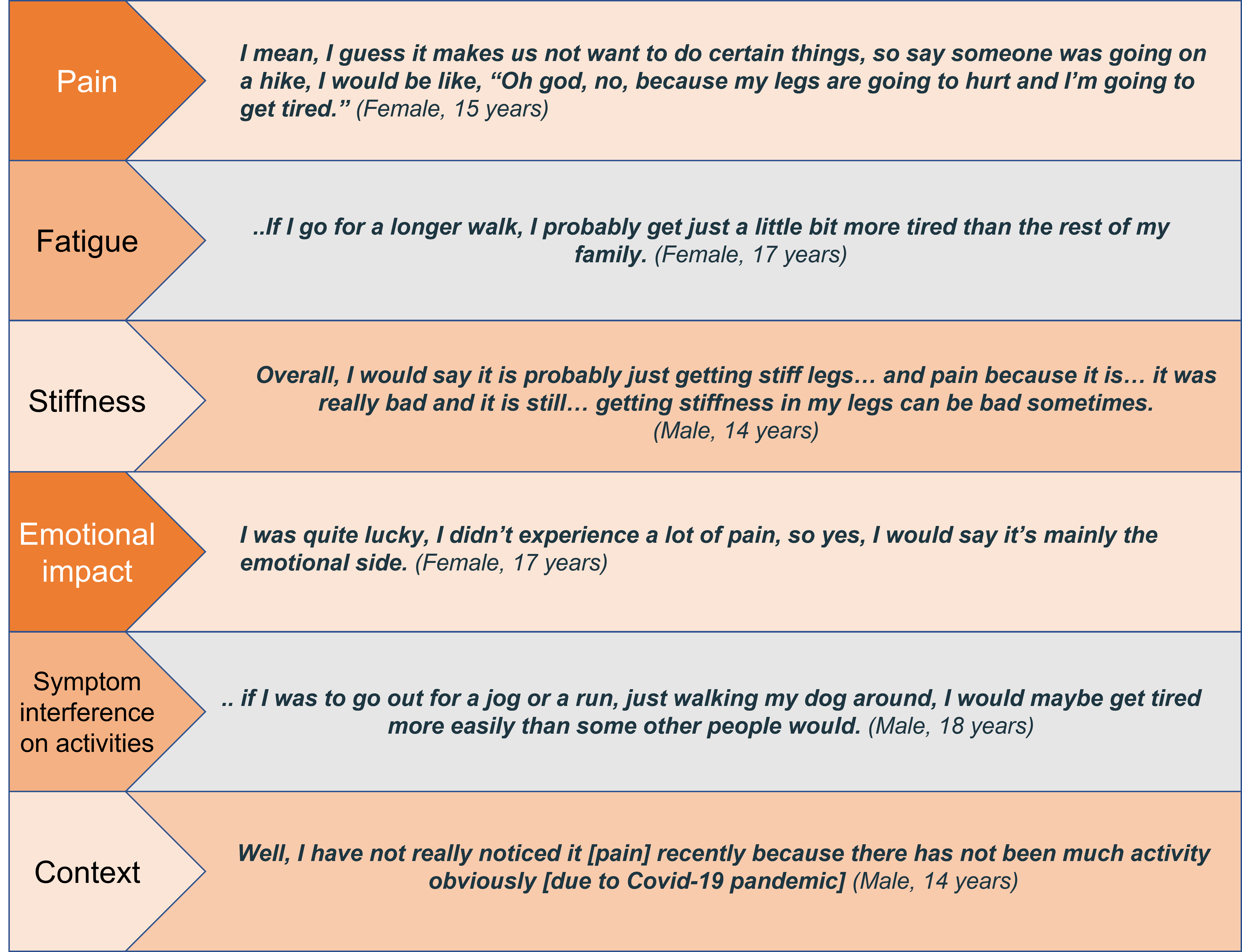

Supplement: S1 Fig — (TIF) [file pone.0295080.s001.tif]
