# Supplementary material for: A patient-centred and multi-stakeholder co-designed observational prospective study protocol: Example of the adolescent experience of treatment for X-linked hypophosphataemia (XLH)
Source: PLoS One. 2024 Jan 19;19(1):e0295080. doi: 10.1371/journal.pone.0295080 (PMC10798437; doi:10.1371/journal.pone.0295080)
Supplement: S1 File — (PDF) [file pone.0295080.s002.pdf]

A patient-centred and multi-stakeholder co-designed observational prospective study protocol: Example of the adolescent experience of treatment for X-linked hypophosphataemia (XLH)

Vrinda Saraff[1 2], Annemieke M. Boot[3], Agnès Linglart[4], Oliver Semler[5], Pol Harvengt[6], Angela Williams[7], Karen M.A. Bailey[8], Fiona Glen[8], Elin Haf Davies[9], Sue Wood[7], Stephen Greentree[7], Angela J Rylands[7]

[1]Department of Paediatric Endocrinology and Diabetes, Birmingham Women's and Children's Hospital NHS Trust, Birmingham, United Kingdom; [2]Institute of Applied Health Research, University of Birmingham, Birmingham, United Kingdom; [3]University Medical Center Groningen, University of Groningen, The Netherlands; [4]Assistance Publique Hôpitaux de Paris, Université Paris Saclay, Bicêtre Paris-Saclay Hospital, Le Kremlin Bicêtre; [5]University of Cologne, Faculty of Medicine and University Hospital Cologne, Department of Pediatrics, Cologne, Germany; [6]XLH Belgium (Belgium XLH Patient Association), Waterloo, Belgium; [7]Kyowa Kirin International, Marlow, United Kingdom; [8]OPEN Health, Marlow, United Kingdom; [9]Aparito, Wrexham, United Kingdom

What is X-linked hypophosphataemia?

X-linked hypophosphataemia (XLH) is a rare, life-long disease caused by changes in a gene. It can affect a person's bones and muscles, and if left untreated, it can become worse as they get older. There is limited information on how the disease affects the health and experiences of adolescents, especially when their bones stop growing, which is an important time as they grow into adulthood. There is a need to better understand the experiences of adolescents with XLH and their caregivers.

What is the My XLH study?

The My XLH study is designed as an 'observational, prospective' study. In this type of study, researchers look at the effect of a risk factor, test, treatment or disease over time without trying to change anything.

The study will be carried out in multiple study centres in the UK and across Europe. In this study, researchers started recruiting on 24 November 2021 and the study is ongoing.

In My XLH, researchers want to better understand the experiences, unmet needs and preferences of adolescents living with XLH before and after their bones stop growing.

Who can take part in the My XLH study?

- This study includes patients who:
  - are aged 12–17 years with a confirmed diagnosis of XLH
  - have been treated with **burosumab** for at least 12 months, and
  - are considered to have reached the end of bone growth by their doctor

What is burosumab?
Burosumab is a medication used to treat XLH, given by injection under the skin. It helps the body manage an important mineral called phosphate, which is needed to make bones and muscles stronger.

What happens in the My XLH study?

- The study was developed together with expert doctors, research and technology specialists, patients with XLH and caregivers
- In this study researchers want to find out about:
  - the lived experience of adolescents before and after their bones stop growing (both with and without burosumab)
  - the role of caregivers supporting adolescents with XLH and the impact on them
  - individual-level changes in wellbeing, emotions, sleep, treatment and daily activities of adolescents with XLH from before to after their bones stop growing

- Researchers collect both quantitative and qualitative data from adolescents with XLH and their caregivers *(see diagram below)* in this study
    - quantitative data refers to number-based, measurable data, which can be statistically analysed, such as severity of symptoms and daily activities (recorded on a smartphone app), as well as heart rate, sleep quality and activity levels (measured by a wearable device)
    - qualitative data are descriptive and interpretable, such as interviews with the patients and caregivers, and questionnaires like the EQ-5D-Y, which asks young people how they feel about their health and life

How is the My XLH study designed?

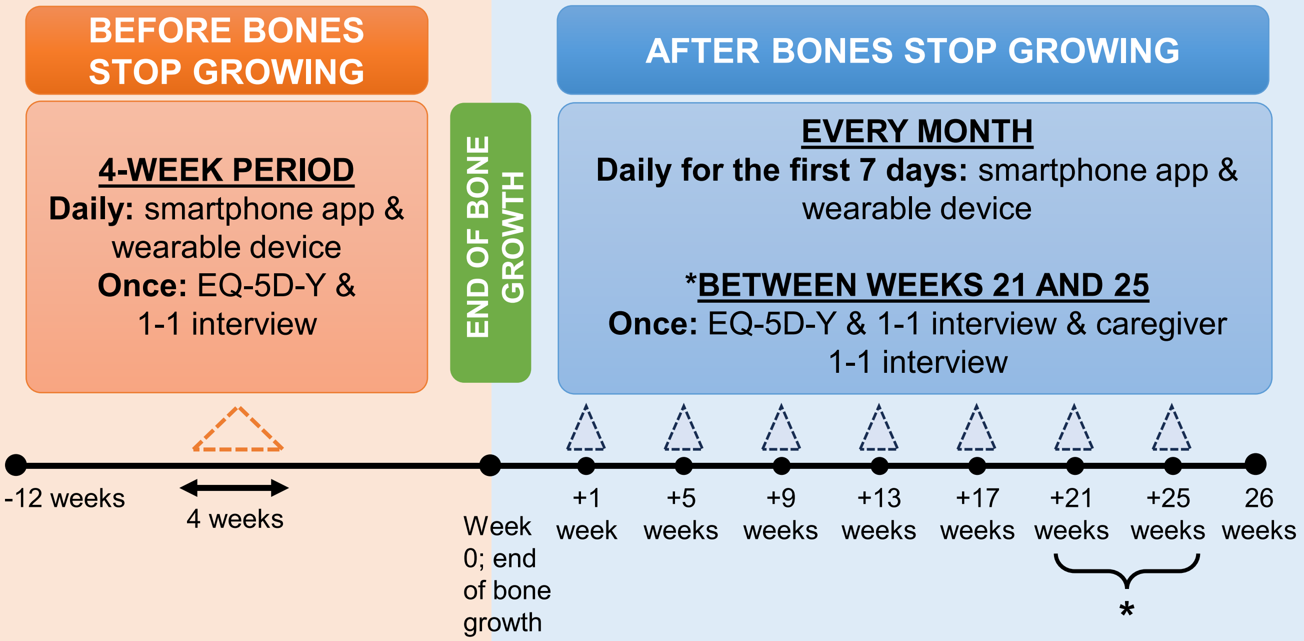

What impact will the study have?

- Involving patients and their caregivers provides valuable insights into their experiences and can inform the care and support they receive
- We expect wearable device data, along with medical records, self-reports and interviews, will enhance understanding of XLH in adolescents, supporting doctors in the decisions they make
- The My XLH study suggests that this kind of inclusive design can be used more widely to assess disease symptoms in a small group of patients with a rare disease at this specific age
